# Combined Thenar and Hypothenar Hammer Syndromes and Raynaud's Phenomenon Successfully Treated with Iloprost

**DOI:** 10.1155/2016/4824929

**Published:** 2016-03-22

**Authors:** Alessandro Ciapetti, Marina Carotti, Marco Di Carlo, Fausto Salaffi

**Affiliations:** ^1^Rheumatology Department, Betsi Cadwaladr University Health Board, Glan Clwyd Hospital, Bodelwyddan, Denbighshire, Wales LL18 5UJ, UK; ^2^Radiology Department, Polytechnic University of the Marche, 60020 Ancona, Italy; ^3^Rheumatology Department, Polytechnic University of the Marche, Jesi, 60035 Ancona, Italy

## Abstract

Thenar and hypothenar hammer syndromes are uncommon conditions characterised by digital ischemia of the hand as a result of repetitive trauma at level of the thenar and/or hypothenar eminence and damage to the radial and/or ulnar arteries, respectively. The symptoms are related to the mechanism of the trauma and a Raynaud's phenomenon can be predominant for a long time. The angiography is the “gold standard” imaging technique which allows to confirm the diagnosis. Therapeutic strategy depends on the type of the lesion and severity of symptoms and includes pharmacological (antithrombotic and thrombolytic drugs) and surgical treatments. The authors present a case of a 53-year-old man, carpenter by profession, with combined thenar and hypothenar hammer syndromes and Raynaud's phenomenon, successfully treated with a short course of intravenous infusion of iloprost.

## 1. Introduction

Thenar and hypothenar hammer syndromes are uncommon conditions characterised by digital ischemia of the hand as a result of repetitive trauma at level of the thenar and/or hypothenar eminence and damage to the radial and/or ulnar arteries, respectively [1–5]. The symptoms are related to the mechanism of the trauma and Raynaud's phenomenon can be predominant for a long time [1, 2, 5, 6]. An isolated hypothenar hammer syndrome (HHS) with involvement of the ulnar artery occurs more frequently than thenar hammer syndrome which is related to the radial artery damage [1, 4, 7]. Nevertheless, a combination of the two syndromes has been reported [7]. The confirmation of diagnosis and the right localization of the vascular occlusion can be obtained by angiography [8, 9]. Therapeutic strategy depends on the type of the lesion and severity of symptoms and includes pharmacological (antithrombotic and thrombolytic drugs) and surgical treatments [3, 4, 7, 10–12].

## 2. Case Report

**Figure 1:**
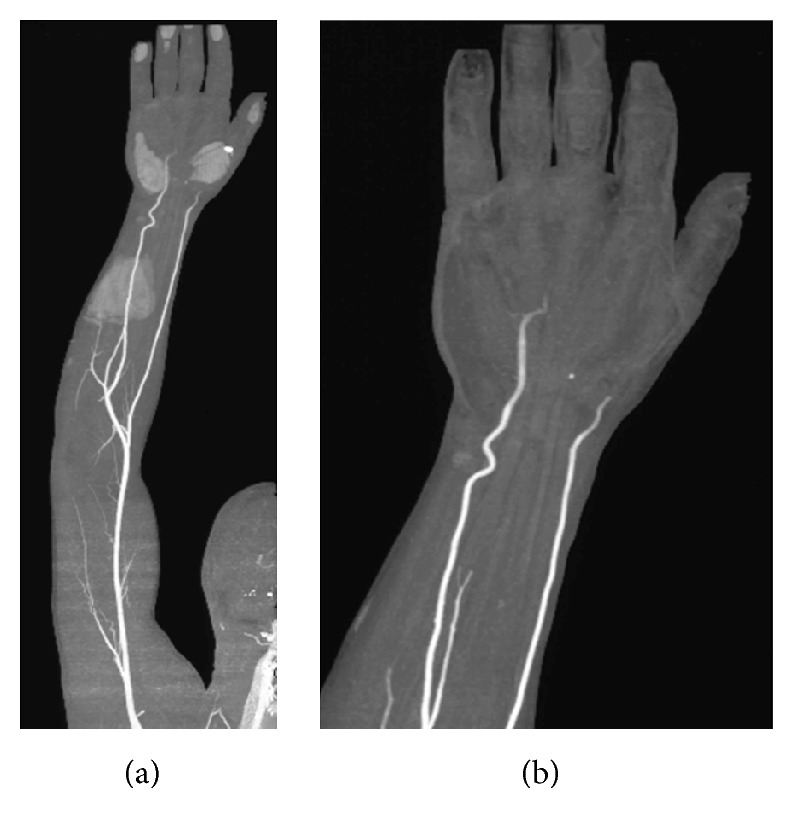
CT angiography of the right upper arm ((a) and (b)). The tridimensional reconstruction (MIP (*Maximum Intensity Projection*) technique) of the arterial circulation showed the interruption of both the radial and ulnar artery flow at level of the thenar and hypothenar eminences and the absence of distal circulation.

A 53-year-old man, carpenter by profession, came to our outpatient department with a 3-month history of decreasing temperature, a white discoloration of the third, fourth, and fifth fingers, and Raynaud's phenomenon of the right hand. The symptoms, initially episodic and then persistent, suddenly appeared after an intensive period of work during which the patient used his dominant hand vigorously. The patient was a free-smoker and denied a previous personal or family history of any cardiovascular diseases. Physical examination did not either show any signs of cardiovascular disease or ischemic changes in the tip of the fingers of his right hand. Nailfold capillaroscopy was negative, whilst color Doppler sonography revealed a decreasing flow of the third digital artery. A 64-slice multidetector computed tomography (CT) angiography of the right upper extremity was performed and showed an occlusion of both the right radial and ulnar arteries at level of the thenar and hypothenar eminences and the absence of distal circulation (Figure 1).

**Figure 2:**
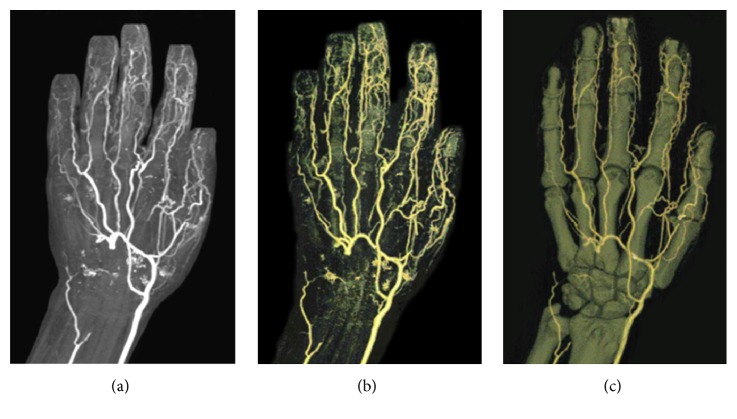
CT angiography of the right upper arm and tridimensional reconstructions ((a)–(c)). The follow-up three months later at the end of an intravenous iloprost course revealed a distal revascularization of the deep palmar arch and common digital arteries.

The patient started an oral pentoxifylline (1.2 g/die) treatment and received an intravenous infusion of iloprost (gradually arriving at a dose of 1.5 ng/kg/min) for 6 consecutive days. Two weeks later at the end of the treatment with iloprost a significantly and persistent improvement of his symptoms was observed. The color Doppler sonography, carried out at the end of the course of intravenous iloprost, showed a clear increase of the flow signals at the level of the third digital artery. A repeated CT angiography, performed three months after the end of the treatment with iloprost, revealed a revascularization of arterial flow of the right hand and showed an improved filling, in particular, of the radial artery and collateral vases (Figure 2).

## 3. Discussion

The term of HHS was initially reported by Conn et al. [13] to describe patients with Raynaud's phenomenon, using their hands as hammer in their occupations, secondary to a repetitive trauma of the ulnar artery. The superficial palmar branch of the ulnar artery is especially susceptible to trauma, because it courses over the hook of the hamate bone in the wrist and a repetitive damage may lead to formation of an aneurysm or thrombosis of the artery [1, 4, 7]. Subsequently, the occlusion of the digital arteries is responsible for development of ischemia symptoms in particular of the third, fourth, and fifth fingers [7]. Different patterns of ischemia can occur and are determined by the anatomic variance of the superficial palmar arch and the small connection between the superficial arch and the radialis indicis or pollicis artery [4, 7].

HHS is more commonly reported among subjects using their hands as hammer in daily occupational activities, but it has been described in athletes as well [7, 14, 15].

The thenar hammer syndrome is much less frequently encountered with respect to the HHS but, usually, patients have similar symptoms and history of repetitive trauma to the palm of their hands. The radial artery is also vulnerable to trauma because of its course over the scaphoid bone.

The “gold standard” imaging techniques to confirm a suspected HHS and/or thenar hammer syndrome are multidetector CT angiography, magnetic resonance imaging angiography, or direct angiography [2, 7–9].

Treatment strategy depends on the severity and spectrum of symptoms and different therapeutic approaches have been adopted [1–4]. Conservative treatment may include changes of daily occupational activities, smoking cessation, and cold avoidance [1, 3]. In case of persistent symptoms a therapy with vasodilatory drugs is recommended [1, 6, 7]. The most common drugs used are calcium channel antagonists, similar to calcium channel antagonists, pentoxifylline, ACE inhibitors, and nitrates [1, 3, 7, 12]. Some authors add the hemodilution to the vasodilators [11]. The efficacy of prostaglandin E1, iloprost, and prostacyclin analog therapy has been documented as well [10, 11]. In a series with a long term follow-up of patients with HHS [11], a five-day regimen of intravenous prostacyclin analog resulted in the complete healing of the subjects suffering of digitals ulcers. The pharmacological therapy with vasodilators could be effectively combined with heparin in the more severe cases to solve the thrombotic obstruction [10]. The persistent severity of symptoms and ischemia may lead to consideration of a surgical approach. Surgical treatment is mainly finalized (a) to remove the source of embolism or a painful mass, (b) to relief nerve compression, and (c) to create a local periarterial sympathectomy [1–4].

Concomitant involvement of the radial and ulnar arteries, as described in our patient, is uncommon. Up to today, only four other reports, involving a total of six patients, described the simultaneous presence of HHS and thenar hammer syndrome [1, 7, 14, 16]. In the first report by Kostianen and Orava [14] the cases of three, well conservatively treated, volleyball players have been described. In the other reports by Neill-Cage et al. [1], McCready et al. [7], and Koulaxouzidis et al. [16] patients underwent a successful surgical management.

In conclusion, in cases of an acute onset of unilateral digital ischemia combined thenar and hypothenar hammer syndromes should be considered, especially in males who use their dominant hand as a hammer. In such cases, as the experience with the above-mentioned patient, a short course of intravenous iloprost can prove successful in restoring the perfusion of soft tissues.
